# Prenatal Diagnosis by Array Comparative Genomic Hybridization in Fetuses with Cardiac Abnormalities

**DOI:** 10.3390/genes12122021

**Published:** 2021-12-19

**Authors:** Katarzyna Kowalczyk, Magdalena Bartnik-Głaska, Marta Smyk, Izabela Plaskota, Joanna Bernaciak, Marta Kędzior, Barbara Wiśniowiecka-Kowalnik, Krystyna Jakubów-Durska, Natalia Braun-Walicka, Artur Barczyk, Maciej Geremek, Jennifer Castañeda, Anna Kutkowska-Kaźmierczak, Paweł Własienko, Marzena Dębska, Anna Kucińska-Chahwan, Tomasz Roszkowski, Szymon Kozłowski, Boyana Mikulska, Tadeusz Issat, Ewa Obersztyn, Beata Anna Nowakowska

**Affiliations:** 1 Department of Medical Genetics, Institute of Mother and Child, Kasprzaka 17a, 01-211 Warsaw, Poland; magdalena.bartnik@imid.med.pl (M.B.-G.); marta.smyk@imid.med.pl (M.S.); izabela.plaskota@imid.med.pl (I.P.); joanna.bernaciak@imid.med.pl (J.B.); marta.kedzior@imid.med.pl (M.K.); barbara.wisniowiecka@imid.med.pl (B.W.-K.); krystyna.jakubow@imid.med.pl (K.J.-D.); natalia.braun-walicka@imid.med.pl (N.B.-W.); artur.barczyk@imid.med.pl (A.B.); maciej.geremek@imid.med.pl (M.G.); jennifer.castaneda@imid.med.pl (J.C.); anna.kutkowska@imid.med.pl (A.K.-K.); pawel.wlasienko@imid.med.pl (P.W.); ewa.obersztyn@imid.med.pl (E.O.); beata.nowakowska@imid.med.pl (B.A.N.); 2 1st Department of Obstetrics and Gynecology Medical University of Warsaw, Plac Starynkiewicza 1/3, 02-015 Warsaw, Poland; marzena@debska.me; 3 Department of Gynecology Oncology and Obstetrics, Medical Centre of Postgraduate Education (CMKP), Czerniakowska 231, 00-416 Warsaw, Poland; ankakucinska@wp.pl (A.K.-C.); tomcior1@gmail.com (T.R.); 4 Clinic of Obstetrics and Gynaecology, Institute of Mother and Child, Kasprzaka 17a, 01-211 Warsaw, Poland; szymon.kozlowski@imid.med.pl (S.K.); boyana.mikulska@imid.med.pl (B.M.); tadeusz.issat@imid.med.pl (T.I.)

**Keywords:** microarray, congenital heart diseases

## Abstract

Congenital heart defects (CHDs) appear in 8–10 out of 1000 live born newborns and are one of the most common causes of deaths. In fetuses, the congenital heart defects are found even 3–5 times more often. Currently, microarray comparative genomic hybridization (array CGH) is recommended by worldwide scientific organizations as a first-line test in the prenatal diagnosis of fetuses with sonographic abnormalities, especially cardiac defects. We present the results of the application of array CGH in 484 cases with prenatally diagnosed congenital heart diseases by fetal ultrasound scanning (256 isolated CHD and 228 CHD coexisting with other malformations). We identified pathogenic aberrations and likely pathogenic genetic loci for CHD in 165 fetuses and 9 copy number variants (CNVs) of unknown clinical significance. Prenatal array-CGH is a useful method allowing the identification of all unbalanced aberrations (number and structure) with a much higher resolution than the currently applied traditional assessment techniques karyotype. Due to this ability, we identified the etiology of heart defects in 37% of cases.

## 1. Introduction

Congenital heart defects (CHDs) are the most common life-threatening birth defects, with an estimated incidence of 0.6% to 0.8%, affecting more than 150,000 in all newborns. The phenotype of CHD is often associated with other anomalies and genetic syndromes. Indications for detailed fetal heart examination, echocardiographic studies include fetal chromosomal abnormality, fetal systemic edema, fetal heart rate disturbance, isolated or multiple cardiovascular defects, and other defects known to be at risk for heart defects (American College of Obstetricians and Gynecologists recommendation (ACOG)). Early detection of CHD in the fetus enables the use of specialized procedures in the perinatal period and in the first days of life. Patients with CHD often require surgical intervention, intensive medical management, and multidisciplinary follow-up. Chromosomal pathogenic abnormalities are detected in 27–28% of children with heart defects coexisting with other birth abnormalities [1] and 3–12% in fetuses with isolated heart defects revealed by ultrasound [2].

The introduction in the 1990s of the chromosome microarray analysis (CMA) method, also known as microarray comparative genomic hybridization, revolutionized cytogenetic diagnostics, enabling detailed results to be obtained in a short time. As a result, in recent years, it has been widely used in prenatal diagnostics, in particular in prenatal diagnostics of congenital heart defects [3,4,5]. The biggest advantage of using microarray over classic cytogenetic and FISH techniques (fluorescence in situ hybridization) is the ability to detect smaller imbalances. Theoretically, classical karyotype analysis by G-banding can detect deletions and duplications greater than 5–10 Mb in size; however, in practice, larger aberrations are often omitted in the analysis. Standard FISH for microdeletion/duplication syndromes usually targets imbalances in the 100–200 kb range but requires clinical features to guide probe selection, which can be a very challenging task for prenatal samples [6].

The use of new methods in diagnostics entails the necessity to change the existing procedures. New standards have been recommended, among others, by Society for Maternal-Fetal Medicine (SMFM) [7], Canadian College of Medical Geneticists (CCMG) [8], European Cytogeneticists Association [9], and American College of Obstetricians and Gynecologists (ACOG) [10].

According to the European Cytogeneticists Association, the American College of Obstetricians and Gynecologists, and the Society for Maternal-Fetal Medicine, chromosomal microarray testing in prenatal diagnosis is recommended for all fetuses, whether in the case of the presence of fetal structural abnormalities or for patients who wished to pursue prenatal diagnosis in the setting of a normal fetal ultrasound. Moreover, this test can be considered in all women undergoing prenatal screening, regardless of age [8,11,12,13,14]. Broader indications for the use of microarrays in prenatal diagnosis are given in the Canadian College of Medical Geneticists guidelines and include congenital abnormalities of the fetus detected by ultrasound or MRI (magnetic resonance imaging) indicating a high risk of unbalanced chromosomal aberration, seemingly balanced hereditary rearrangements in the fetus with diagnosed congenital abnormalities, and apparently balanced de novo rearrangements detected by classical cytogenetics. At the same time, the guidelines emphasize that CMA testing should not be performed in pregnancies with a low risk of chromosomal abnormalities [9].

Based on the above recommendations, copy number variations detected in the array CGH study (comparative genomic hybridization) can be classified as pathogenic, likely pathogenic, likely benign, benign, and variants of unknown significance (VOUS). To interpret the clinical significance of the structural CNVs (copy number variations), many factors should be considered, including its type (deletion/duplication) and size, content of genes mapped in a given region, and parental origin. To determine the origin of aberrations, a routine karyotype analysis by the GTG technique (G-bands after trypsin and Giemsa), FISH, array CGH, or MLPA method (Multiplex Ligation-dependent Probe Amplification) should be performed on the fetus’s parents depending on the type and size of the variant, and on the available methods. Interpretation of the clinical significance of unknown CNVs is often very complicated and there is no single general rule or algorithm for the interpretation of test results.

Due to the unknown clinical impact, the greatest diagnostic challenges are VOUS changes found in prenatal testing. Therefore, in accordance with the recommendations issued by, among others, the European Cytogeneticists Association, these aberrations are not reported in prenatal results [9]. Whereas, to minimize the reporting of uncertain findings, the CCMG indicates that variants of unknown clinical importance smaller than a 500 kb deletion or a 1 Mb duplication should not be shown in the prenatal reports [9].

VOUS, in contrast to likely pathogenic aberrations, does not include genes of known pathogenicity and does not occur in a few cases of the general population as likely benign. The number of VOUS will decrease as their significance is published more in medical literature. The VOUS frequency remains quite variable. For example, Song T. et al. reported variants of unknown significance (VOUS) in 14/190 (7.37%) cases [15], Lee M. et al. in 4/32 (12.5%) [16], Yan Y. et al. in 4/76 (5.3%) [17], and Wu et al. in 2.9% (3/104) of cases [18]. This difference can be explained by the different resolution of the microarray platform used by the authors and the differences in interpreting the meaning of the same VOUS [14]. Furthermore, a variant of unknown significance may in fact be pathogenic but has been classified as VOUS because of insufficient data to determine its real pathogenic significance. Therefore, a better understanding of VOUS is needed to provide adequate prenatal genetic counseling [15].

Using the array CGH method to diagnose congenital heart defects in fetuses enables more information on potential genetic causes and the correlation between the genotype and phenotype to be obtained. Additionally, this method can determine the origin of the detected change in parents [19]. Chromosomal causes of CHD include chromosome aneuploidies, like trisomy 21, 18, 13, and structural copy number variations. The most common cardiac defects associated with trisomy 13 and trisomy 18 are atrial septal defect (ASD), ventricular septal defect (VSD), and patent ductus arteriosus (PDA) [20,21,22]. There is a wide spectrum of CHD in patients with trisomy 21. Atrioventricular septal defect (AVSD) is the most frequent CHD in patients with T21 (30–60%), followed by ASD (16–21%) and VSD (14–27%), tetralogy of Fallot (TOF) (2–11%), patent arterial duct (6%), and aortic coarctation (CoAo) (0.3%). Some of the CHD lesions could be isolated or combined with other types of CHD [23,24].

The spectrum of CHD-related CNVs ranges from recurrent microdeletion and microduplication syndromes, such as 22q11.2 Deletion Syndrome, 22q11.2 Duplication Syndrome, and Williams–Beuren syndrome, which are associated with a distinct clinical recognizable phenotype, to rare CNVs, flanked by unique breakpoints [18]. Heart defects indicate specific phenotypic features in microdeletion syndromes, e.g., in 75% of the cases with deletion syndrome 22q11.2, the most common defects are TOF, arterial atresia pulmonary, VSD, and common arterial trunk (CAT); in the Williams syndrome (interstitial deletion 7q11.23), supravalvular aortic stenosis (SVAS) is a characteristic; and in the 1p36 deletion syndrome, the most common defects are cardiomegaly, Ebstein syndrome, and ASD. Heart defects, e.g., mitral valve prolapse, also occur in 3–50% of patients with Fragile X syndrome [25,26].

The use of CMA in the diagnosis of prenatally diagnosed CHD is described in the article by Jansen et al. (2015) [24]. They summarized 13 publications with a total of 1131 microarray cases. Excluding aneuploidy and 22q11.2 deletion, clinically significant CNVs were observed on average in 7.0% (5.3–8.6%), 0.3–6.6% in “isolated” CHD, and 6.6–12% in CHD with other defects. Remaining aberrations of unknown clinical significance were identified in an additional 3.4% of fetuses with CHD. Thus, when using the microarray method, about a 14% probability of detecting an aberration considered clinically pathogenic and variants of unknown significance can be expected: 4% 22q11.2 deletion, 7% other pathogenic CNV, and 3% VOUS. The only caveat is that pathogenic CNV included both these CHD-related variants and “random” findings relevant to, for example, neurological development. Postnatal studies confirm a similarly high and increasing percentage of pathogenic CNVs in isolated malformations and coexisting CHD with another malformation, as well as a high percentage of VOUS. Due to the growing knowledge of the importance of CNVs, previously obtained results of unknown significance should be re-analyzed for pathogenicity [2].

In this study, we present the results of the chromosomal microarray analysis in a cohort of 484 cases with prenatally diagnosed congenital heart diseases by fetal ultrasound scanning (256 isolated CHD and 228 CHD coexisting with other malformations).

In clinical reports, we included only pathogenic and likely pathogenic aberrations. We identified pathogenic aberrations and likely pathogenic genetic loci for CHD in 165 fetuses and, additionally, 10 CNVs of unknown clinical significance.

## 2. Materials and Methods

Samples were received over a 5-year period from the Institute of Mother and Child in Warsaw and two others centers in Poland. The inclusion criteria were either multiple abnormalities included in CHD or isolated heart abnormality observed on ultrasound for which invasive testing and genetic analysis is advised. Informed consent was obtained from all patients included in the study. We received biological material samples from fetuses and their parents after signing informed consent, using protocols approved by the Institute of Mother and Child in Warsaw.

### 2.1. Sample Types and DNA Isolation

Amniotic fluid (AF) samples, chorionic villi samples (CVSs), and fetal blood samples were received. In all cases, backup cultures were carried out for further DNA requirements and for conventional karyotype analysis. Genomic DNA was instantly isolated from fresh material. When required, DNA was isolated from cultured cells (AF samples and CVSs only). Villi from CVSs were separated from maternal tissue under a microscope to minimize maternal cell contamination (MCC). Two to four villi were provided for DNA extraction. Between 2 and 5 mL of amniotic fluid sample were provided for DNA extraction. AF samples were centrifuged. Genomic DNA was extracted using the DNA isolation kit (Sherlock A & A Biotechnology, Gdynia, Poland) following the manufacturer’s recommendations. For CVSs, incubation at 56 °C with 20 µL of proteinase K, water, and tissue lysis buffer (Buffer L1.4) was performed for at least 1 h for efficient digestion and lysis of the complete sample. For AF samples, incubation at 56 °C with 20 µL of proteinase K, water, and lysis buffer (Buffer L1.4) was performed for 45 min. DNA isolation was performed according to the manufacturer’s instruction.

### 2.2. Genomic Array Platform (Array Comparative Genomic Hybridization (Array CGH) Analysis and Interpretation)

The array CGH was performed using a 60K microarrays -8x60K from Oxford Gene Technology (CytoSure ISCA, v3, Oxford, UK). The array used in this study contains 51 317-mer oligonucleotide probes covering the whole genome with an average spatial resolution of 60 kb. For all stages of the test, DNA denaturation, labeling, and hybridization were performed in accordance with the attached manufacturer’s instructions. Whole gene DNA was labeled for 2 h using the CytoSure Labeling Kit (Oxford Gene Technology, Oxford, UK), without enzyme digestion. Hybridization was performed for 24 to 48 h in a rotary oven (Agilent Technologies, Santa Clara, CA, USA) at 65 °C. An Agilent 1 and 2 set of washing solutions were used to wash the arrays. The arrays were scanned on an Agilent Technologies microarray scanner, and then the signal intensities were calculated using Feature Extraction software (Agilent Technologies). Scanned images were quantified using Agilent Feature Extraction software (V10.0). All genomic coordinates are based on a reference genome (NCBI37/hg19). The analysis of the obtained data was performed using the CytoSure Interpret Software (Oxford Gene Technology, Oxford, UK) and the circular binary segmentation algorithm. The calling thresholds are the deviation of the cyclic binary segmentation (CBS) segment from a zero logarithmic ratio of +0.30 for duplication and −0.5 for deletion. Then, results were classified using the CytoSure Interpret Software (Oxford Gene Technology). All quality control measures were monitored using CytoSure Interpret Software (Oxford Gene Technology).

The microarray used in this analysis does not contain SNP probes nor does it detect polyploidy, inversion, balanced translocation, and regions of absence of heterozygosity.

### 2.3. CNV Classification

The clinical relevance of copy number variants should be considered individually using the general CNV classification. In our study, we used five categories of classification of detected aberrations: pathogenic, likely pathogenic, variants of unknown significance (VOUS), likely benign, and benign.

1. Pathogenic aberrations: CNV is classified as pathogenic if it is a big aberration of several Mb, or it is one of the recurrent genomic disorders and known microdeletion/microduplication syndromes, or it contains known genes involved in a particular pathology and was previously described in specific clinical disorders.
2. Likely pathogenic aberrations: CNVs that have not yet been described or have been described infrequently and contain some gene/genes whose function is known and may be responsible for the patient’s clinical features. Likely pathogenic aberrations overlap genomic regions that are associated with intellectual disability, dysmorphic features, and/or congenital malformations.
3. Variants of unknown significance (VOUS): This category includes all CNVs that have no clearly defined clinical relevance at the time the test result was released. These aberrations have not been reported in prenatal results, because the function of genes in this region is unknown or difficult to associate with the ultrasound abnormalities. These aberrations were not verified in parents, although it might help to further interpret them as likely benign or likely pathogenic.
4. Likely benign aberrations: CNVs that have not been described but are present in healthy people and have only been described in a few cases in the general population but do not represent a common polymorphism. CNVs interpreted as likely benign were not reported.
5. Benign aberrations: These CNVs do not affect the phenotype (polymorphisms found in the general population), which include aberrations in the region of segmental duplication, aberrations that do not contain genes, aberrations in areas containing dose-insensitive genes often recurring in the Polish population, and aberrations known as copy number variants described in the Database of Genomic Variants database (http://dgv.tcag.ca/dgv/app/home accessed on 6 September 2021) (track: DGV Gold Standard Variants). Known polymorphic CNVs were interpreted as benign and not reported.

All detected copy number variants (CNVs) were systematically evaluated for clinical significance by comparing them with those in the scientific literature and available databases: OMIM (http://www.ncbi.nlm.nih.gov/omim accessed on 6 September 2021), Clinical Structural Variants (https://www.ncbi.nlm.nih.gov/dbvar/ accessed on 6 September 2021), Database of Genomic Variants (http://projects.tcag.ca/variation/ accessed on 6 September 2021), Ensembl (https://www.ensembl.org/index.html accessed on 6 September 2021), and DECIPHER (http://decipher.sanger.ac.uk/ accessed on 6 September 2021).

## 3. Results

**Figure 1:**
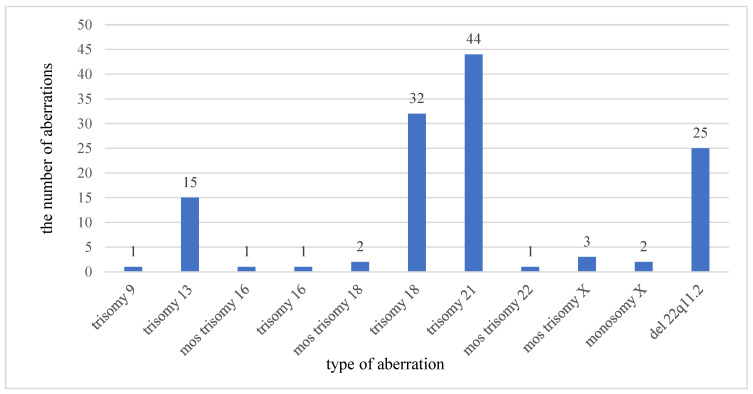
Frequency of chromosomal aneuploidy and microdeletion 22q11.2 in all 176 abnormal results (mosaic trisomy (mos), deletion (del)).

In this study, we report the detection rate of chromosomal microarray analysis among 484 fetuses with congenital heart defects in Poland. Chromosomal aberrations were found in 176 cases (74 in isolated CHD and 102 in CHD coexisting with other malformations). Chromosomal aneuploidy was detected in 102 cases and the most common 22q11.2 microdeletion syndrome was detected in 25 cases (Figure 1). The most common aneuploidy are trisomy of chromosome 21 (Down Syndrome), 18 (Edwards syndrome), 13 (Patau syndrome), and monosomy X (Turner syndrome). Heart defects occurred in 91% of cases with Down Syndrome, 79% in cases with Edwards Syndrome, and 67% in cases with Patau Syndrome (Table 1). The most frequent heart disease was tetralogy of Fallot observed in 80% of fetuses whereas VSD was observed in 12% and HLHS (hypoplastic left heart syndrome) in 8% of fetuses with 22q11.2 microdeletion syndrome.

Other pathogenic structural aberrations were found in 22 cases (Table 2). Likely pathogenic structural aberrations were found in 18 cases (Table 3) and VOUS structural aberrations were found in 9 cases (Table 4).

## 4. Discussion

Rapid unbalanced chromosomal abnormalities detection by array CGH techniques performed in all fetuses with abnormal ultrasound findings is a good tool used as a first diagnostic screening test, as it permitted diagnosis of aneuploides and structural aberrations in 37% of the cases of our cohort. Our detection rate was higher than the overall rate described in recent publications on prenatal CMA (2.7% in Lee C.N. et al. 2012 [27], 3.3% in Fiorentino F. et al. 2011 [28], 4.2% in Breman A., et al. 2012 [29], and 12% in Rooryck et al. 2013 [30]). This result showed that by selecting indications that are clinically relevant, based primarily on the combination of abnormal ultrasound findings, CMA produces a greater “yield” of clinically relevant CNVs.

The microarray method not only provides information about the presence of CNV, but it is also possible to identify the chromosomal mosaicism. We identified three types of mosaic trisomies of chromosomes 16, 18, and 22 (fetus 598, 713, 981, 1286, and 1409). Chromosomal mosaicism is defined as the presence of two or more distinct cell lines in an individual. Prenatally, chromosomal mosaicism most commonly affects only the placenta but sometimes extends to the fetus. The clinical consequences of chromosomal mosaicism identified during invasive prenatal diagnosis can be difficult to predict, ranging from no apparent phenotypic effect to early fetal lethality. Values for detection chromosomal mosaicism are a log2 ratio from 0 up to +0.3 or from 0 down to −0.5. We identified the smallest mosaicism at a level of 11% in a fetus with VSD (mosaic trisomy 22). Cell culture in conventional karyotype may promote the in vivo selection of euploid over aneuploid cells, which has been reported to increase with the age of the culture. This study demonstrates that CMA is a very sensitive diagnostic tool, and it can identify chromosomal mosaicism at a lower level than routine conventional karyotype analysis by the GTG technique and FISH method performed on amniotic fluid cells after cell culture.

In our study, a total of 60 different aberrations were found in 176 out of 484 fetuses with CHD. The first group contained 33 aberrations considered to be clinically pathogenic (e.g., pathogenic for VSD, AVSD, TOF) (Figure 1 and Table 2). This group included aneuploidies and imbalances greater than 5 Mb in size identified in 9 fetuses (not seen in standard cytogenetic studies). The most common found were aneuploidies (~58%): trisomy 21 in 44 cases, trisomy 18 in 32 cases, and trisomy 13 in 15 cases.

The second group consisted of 18 likely pathogenic CNVs for CHD (Table 3). The deletions and duplications were classified as likely pathogenic CNVs when they contained candidate genes that may contribute to the abnormal phenotype. Aberrations inherited from healthy/normal parents require a thorough analysis and detailed description of the genetic burden in the family. The qualification of an aberration as likely pathogenic should also take into account the uniparental disomy, incomplete gene penetration, and other genetic factors. In our research, likely pathogenic aberrations accounted for 36.7% (18/49) of all identified CNVs and 10% (18/176) of all identified aberrations. The origin of these aberrations was determined in 14 cases. In total, 14% (2/14 cases) of likely pathogenic aberrations arose *de novo*, while the remaining 36% (5/14 cases) were inherited from a normal mother, and 50% (7/14 cases) from a normal father and were identical like in the fetus. Here, we present aberrations that contain genes that may be responsible for fetal CHD. In case 383, we identified duplication of 1q32.1 (approximately size 1.22 Mb) and 3p26.3 deletion (approximate size of 1.99 Mb) in the fetus with ARSA. Duplication in 1q32.1 includes four genes: *DDX59* (OMIM: 615464), *KIF14* (OMIM: 611279), *ZNF281*, and dose-sensitive *NR5A2* gene (OMIM: 604453). KIF14 is a member of the kinesin superfamily of microtubule-associated motors that play important roles in intracellular transport and cell division (OMIM: 611279). Mutations in the *KIF14* gene are described in patients with Meckel Syndrome-12 (OMIM: 616258) and Microcephaly-20 (OMIM: 617914). Affected children or fetuses with Meckel syndrome may also have abnormalities affecting the head and face (craniofacial area), liver, lungs, heart, and genitourinary tract. Heart abnormalities may include atrial and ventricular septal defects (ASDs and VSDs) and patent ductus arteriosus or other more complex malformation. We did not find a similar duplication in the ClinGen and DECIPHER databases. Only the ClinVar database showed six pathogenic aberrations (approximately 3–5 Mb in size). The 3p26.3 deletion includes two genes: *CHL1* (OMIM: 607416) and the dosage-sensitive *CNTN6* gene (OMIM: 607220). Parental array CGH showed that identical aberrations were detected in normal mother.

We detected a 3.26 kb duplication in case number 978 with mitral regurgitation. Unfortunately, in our study, parental samples were not available for verification. Duplication included exon 16–20 of the *KMT2A* gene. This gene encodes a DNA-binding protein that methylates histone H3 (H3K4) and positively regulates the expression of target genes, including multiple *HOX* genes. Mutation in *KMT2A* has been associated with Wiedemann–Steiner syndrome (WDSTS, OMIM: 159555) with autosomal dominant inheritance. The ClinVar database showed pathogenic mutations in the *KMT2A* gene, also known as MLL. Features of WDSTS patients include intellectual disability, craniofacial defects, and skeletal and heart defects. De novo frameshift mutation (p.Glu390Lysfs∗10) in the *KMT2A* gene was described in a 10-year-old boy with congenital heart disease (ventricular septal defects) [31].

In case 1009 with hypoplastic left heart syndrome, we detected duplication 2p13.1 (approximately 430 kb in size). Duplication in 2p13.1 contains the *ACTG2* gene (OMIM: 102545). The *ACTG2* gene provides instructions for making a protein called γ-2 actin, which is part of the actin protein family. Actin proteins are highly conserved proteins that are involved in various types of cell motility and in the maintenance of the cytoskeleton. Parental array CGH analyses showed the same duplication in chromosomal region 2p13.1 in the phenotypically normal father. We did not find a similar duplication in the ClinGen, DECIPHER, or ClinVar database.

In case number 2155, an 8.33 kb duplication of chromosome 8p23.1 was found in a fetus with ventricular septal defect and pulmonary stenosis. The duplication included exon 2 of the *GATA4* gene (OMIM: 600576). This gene encodes a member of the GATA family of zinc finger transcription factors. Mutations in this gene have been associated with cardiac septal defects as well as reproductive defects. Unfortunately, parental samples were not available for verification. The ClinVar database showed six intragenic duplications (approximately size 50 kb) in patients with ASD.

Interestingly, 202 kb deletion of chromosome 5q35.3 was detected in the fetus with the tetralogy of Fallot (case number 5528). Aberration includes exons 4–14 of the dose-sensitive *CNOT6* gene (OMIM: 608951), *SCGB3A1* (OMIM: 606500), and *FLT4* (OMIM: 136352). Pathogenic variants in the *FLT4* gene have been reported in patients with congenital heart disease type 7 (CHTD7, OMIM: 618780) inherited as an autosomal dominant with incomplete penetrance. This disorder is mainly characterized by tetralogy of Fallot but also includes right-sided aortic arch, absent pulmonary valve, and other cardiac abnormalities [32]. *FLT4* encodes vascular endothelial growth factor receptor 3 (VEGFR-3), which regulates the development and maintenance of the lymphatic system, and it is one of three main cell surface receptors for vascular endothelial growth factors. Additionally, a deletion involving the *FLT4* gene was described in a patient with the tetralogy of Fallot [33]. CGH analysis of parental samples showed that this aberration was inherited from a normal mother.

The technology based on the microarray CGH microarray method allows the identification of the whole spectrum of CNV sizes, from aneuploidy to very small submicroscopic aberrations. In addition to pathogenic, likely pathogenic, or benign aberrations, this method identifies many variants of unknown clinical significance. Those deletions and duplications were classified as VOUS for CHD based on the following criteria: CNVs that have no clearly defined clinical relevance at the time the test result is released; includes a gene or genes that have an unknown effect on the identified fetal defect (Table 4). In our study, VOUS were detected in 9 fetuses, representing 1.8% of all tested fetuses with CHD or 5.1% of fetuses in which CNV was found.

Ethics related to genetic counseling and prenatal diagnosis is complex. The greatest challenge for genetic diagnosis is the VOUS found in prenatal testing. Therefore, it is very important to better understand clinically relevant genetic variants. Additional studies are necessary to determine the possible pathogenic effect of VOUS changes. Very often, the use of different techniques does not always provide data on VOUS; only association studies are necessary to determine their exact pathogenic potential. As the potential benefits have to be weighed against the possible risks, it is currently recommended that only pathogenic or likely pathogenic CNVs be disclosed to parents. Reporting VOUS aberrations in the results can be associated with significant stress and anxiety for parents [34].

In all cases, where VOUS was detected, due to the informed consent, parents were not informed about the variant; therefore, the parental origin could not be verified. One such example is case 1278, which was referred for testing because of excessive heart rotation, invisible stomach, and bilateral kidney pyelectasis on prenatal ultrasound. We identified a 522 kb duplication of chromosome 1p36.32. This duplicated region contains the *PRDM16* gene (OMIM: 605557). Cardiac malformations and cardiomyopathy are one of the most common congenital abnormalities caused by a chromosome 1p36 deletion that affects approximately one in 5000 newborns. PRDM16 is a zinc finger transcription factor that controls the development of brown adipocytes in brown adipose tissue and the cell fate between muscle and brown fat cells. *PRDM16* is localized in the critical region for cardiomyopathy defined by Gajecka et al. [35] and deletions encompassing this gene were described in patients with heart muscle disease (left ventricular noncompaction or cardiomyopathy). Moreover, *PRDM16* is expressed in the human artery, nerve, thyroid, stomach, and kidney (https://www.gtexportal.org/home/gene/ENSG00000142611 accessed on 6 September 2021).

Case 674 was referred due to tricuspid insufficiency. A 440 kb duplication of chromosome 2p16.3 was detected. The duplicated region contains the *FBXO11* gene (OMIM: 607871). This gene encodes a member of the F-box protein family, which is characterized by approximately 40 amino acid motifs, the F-box. *FBXO11* variants were also identified in human cancers, such as colon, lung, ovary, and head and neck tumors. In mice, a homozygous mutation of *FBXO11* results in cleft palate defects, facial clefting, and dysmorphic features. This VOUS duplication most likely was not the cause of the phenotype.

In case 1165, the array showed a 335 kb duplication of chromosome 9q21.32q21.33 in a fetus with ventricular septal defect. The duplication of chromosome 9q21.32q21.33 included one OMIM gene, *SLC28A3* (OMIM: 608269). *SLC28A3* encodes Solute Carrier Family 28 Member 3, which plays a role in multiple cellular processes, including neurotransmission, vascular tone, adenosine concentration in the vicinity of cell surface receptors, and transport and metabolism of nucleoside drugs.

Case number 2093 involved a 158 kb duplication of chromosome 10q26.12 in a fetus with tetralogy of Fallot. This duplicated region contains the *WDR11* gene (OMIM: 606417). WDR11 is a member of the WD repeat-containing protein family. Heterozygous mutations in the *WDR11* gene were described in patients with congenital idiopathic hypogonadotropic hypogonadism (IHH).

In recent years, an additional diagnostic tool introduced to understand the molecular mechanisms regulating the growth of the heart is whole-exome sequencing (WES). As a result, it was possible to identify numerous transcriptional regulators, signaling molecules, and genes important for normal cardiac morphogenesis. The WES studies conducted so far have shown many candidate genes that are potential pathogenic variants of congenital heart defects. For example, in the case of atrial septal defect (ASD) and ventricular septal defect (VSD), candidate genes can be counted: *NKX2-5*, *GATA4*, *TBX20*, *MYH6*, and *TBX5*, while the pathogenesis of atrioventricular septal defect (AVSD) involves genes, such as *PTPN11*, *KRAS*, *SOS1*, *RAF1*, and *CRELD1*. Additionally, in the available literature, new information about new pathogenic variants in known genes associated with CHD can be found, e.g., mutations in the *TLL1* gene (p. I236V) are assessed as likely pathogenic in an atrial septal defect. Similarly, a mutation in the *MYH11* gene (p. V321M) is classified as pathogenic in the presence of patent ductus arteriosus (PDA) [36,37,38].

Whole-exome sequencing (WES) in children with congenital heart disease diagnoses an additional 20–40% of cases with normal GTG and aCGH results. Therefore, it is reasonable to use this method when the chromosomal aberration is not found [39]. For the last few years, WES prenatal testing has been used as a diagnostic element in many laboratories around the world and is becoming more and more popular among pregnant women [40,41]. The studies conducted so far show that the detection rate for pathogenic variants in fetuses with congenital heart defects found in USG is 10–30% [42]. The use of the WES technique in the diagnosis of CHD provides an additional opportunity to diagnose the cause of the defect found in the fetus.

All the presented results confirm the fact that ultrasound diagnosis of the heart defect is an indication for invasive prenatal diagnosis in order to determine the fetal karyotype. There is a high risk of chromosomal aberration, especially in cases where congenital heart defect and other fetal defects are diagnosed.

This study highlights the utility of CMA in fetuses with CHD. The use of the microarray method significantly increases the detection rate of pathogenic CNVs, which are the cause of congenital heart defects.

## Figures and Tables

**Table 1:** Specific heart defects of our cases with common aneuploidies.

Aneuploidy	Heart Defects in Ultrasound
Trisomy 21	AVSD, VSD, TOF, ASD
Trisomy 18	VSD, AVSD, DORV
Trisomy 13	VSD, ASD
Monosomy X	VSD

(Atrial septal defect (ASD), atrioventricular septal defect (AVSD), double outlet right ventricle (DORV), tetralogy of Fallot (TOF), and ventricular septal defect (VSD)).

**Table 2:** Pathogenic structural aberrations found in our cases.

Patient	Prenatal Diagnosis	Aberration (Inheritance—If It Has Been Identified)	Size
1166	Cleft palate, VSD, foot deformation	1p36.33p36.22(779733_9620926)x1,5p15.33(22149_2274755)x3	8.8 Mb; 2.2 Mb
254	cardiomegaly	1q21.1q44(142491666_246928498)x3	104 Mb
2054	HLHS	2p25.3(21191_3062258)x1,12q24.13q24.33(113023613_133773393)x3	3 Mb; 21 Mb
1220	TOF	1q42.12q44(226703815_249203359)x3, 9q34.3(138907844_141018976)x1	22.2 Mb; 2.1 Mb
653	VSD	3p22.2(37646228_38961056)x1,3q24q25.32(145448788_158594702)x1	1.3 Mb; 13 Mb
1214	AVSD	3p24.1p22.3(27228808_33971880)x1	6.7 Mb
551	VSD	5p15.33p12(22149_45362363)x3	45 Mb
13	TOF	6q25.3q26(156813910_162033274)x3 dn	5.2 Mb
322	VSD	6q26q27(163436214_170847447)x1 dn	7.4 Mb
589	ASD	7p14.3p14.1(31773017_42738664)x1	11 Mb
1478	VSD	8p23.1(7113656_12454089)x1	5.34 Mb
784	AVSD, TOF	8p23.1p21.3(6224261_21242145)x1	15 Mb
1258	Cleft palate, AVSD	8p23.3p21.2(191605_24918147)x3,9p24.3q21.32(204090_84386182)x3	27 Mb; 84 Mb
1006	AVSD	8p23.3p23.1(191605_12454089)x1 mat, 18p11.32p11.31(149089_7094765)x3 mat	12 mb; 6 Mb
983	VSD	10q11.22q26.3(46426869_135404550)x3	89 Mb
1262	ASD	11p15.5p11.2(113082_46371104)x3	46 Mb
173	VSD, CoAo	12p13.33p11.1(100698_34647463)x3	34.5 Mb
1148	CoAo	13q21.1q21.32(57950814_67755631)x3 dn	9.8 Mb
1065	IUGR, VSD, ARSA	14q24.3q32.31(79087813_102919927)x1	23.8 Mb
1306	VSD	15q11.1q11.2(20686203_23586302)x1,(18)x3	2.9 Mb; 80.7 Mb
1995	cardiomegaly	16p11.2q24.3(34202297_90252496)x3	56 Mb
404	HLHS	17p13.3p13.2(1656_5534353)x1	5.53 Mb

In some cases, inheritance was not determined due to a lack of contact with parents (aberrant right subclavian artery (ARSA), aortic coarctation (CoAo), hypoplastic left heart syndrome (HLHS), and intrauterine growth restriction (IUGR); inheritance: maternal (mat), paternal (pat), *de novo* (dn)).

**Table 3:** Likely pathogenic structural aberrations found in our research group.

Patient	Prenatal Diagnosis	Aberration (Inheritance—If It Has Been Identified)	Size
383	ARSA	1q32.1(197684386_198909224)x3 mat,3p26.3(69430_2062244)x1 mat	1.22 Mb; 1.99 Mb
1009	HLHS	2p13.1(73763801_74194368)x3 pat	430 kb
698	VSD, Dandy-Walker syndrome	2p15(61632727_62017908)x3 pat	385 kb
986	AVSD	2p16.3(50880241_50949412)x1 (Additionally, this patient had trisomy of chromosome 13)	64 kb
1736	VSD	3p12.3(77192875_79219598)x1 pat	2 Mb
608	AVSD	4p15.32(16064173_16813206)x3 mat	989 kb
395	AVSD	5q35.3(177068821_178058571)x3 pat	900 kb
447	HLHS	5q35.3(177956887_178917587)x3 mat	1 Mb
2155	VSD, ARSA	8p23.1(11550005_11558331)x3	8.3 kb
978	mitral regurgitation	11q23.3(118363939_118367204)x3	3.26 kb
888	AVSD	14q23.3q32.33(67146824_107287708)x3 dn, Xp21.1(31699053_31805802)x1 dn	40 Mb; 106 kb
1199	AVSD, HLHS	16p11.2(28318123_29182200)x3 pat	864 kb
1122	AVSD	17p12(14111754_14423151)x3,17p12(14911841_15322595)x3	311 kb; 411 kb
948	HLHS	17q12(34652173_36290311)x1 pat	1.68 Mb
851	HLHS	18q11.1(18542080_18672140)x1 pat	130 kb
1658	VSD	Xp22.2(11600766_12080374)x3 mat	479 kb
2195	VSD	Xq28(153324080_153362472)x3 dn	30 kb
5528	TOF	5q35.3(179950554_180152423)x1 mat	202 kb

In some cases, inheritance was not determined due to a lack of contact with parents.

**Table 4:** VOUS structural aberrations.

Patient	Prenatal Diagnosis	Locus	Size	Gene	Associated Anomalies
584	cardiac ectopy	13q13.3(37145323_37351415)x3	206 kb	*SERTM1*	Serine Rich and Transmembrane Domain Containing 1 protein with high expression in cancer tissue.
674	tricuspid valve regurgitation	2p16.3(48059806_48500445)x3	440 kb	*FBXO11*	*FBXO11* mutations were also identified in human cancers, such as colon, lung, ovary, and head and neck tumors. In mice, a homozygous mutation of *FBXO11* results in cleft palate defects, facial clefting, and dysmorphic features
765	atrioventricular septal defect (AVSD)	11q22.1(101436248_101756583)x3	320 kb	*ex 1 TRPC6*	*TRPC6* encodes Transient Receptor Potential Cation Channel Subfamily C Member 6. Mutations in the *TRPC6* cation channel causes familial focal segmental glomerulosclerosis. TRPC6 is a known factor in cardiac hypertrophy and heart failure.
1045	Ebstein Syndrome	21q11.2(15824276_16137741)x3	313 kb	*SAMSN1*	*SAMSN1* is a member of a novel gene family of putative adaptors and scaffold proteins containing SH3 and SAM (sterile α motif) domains. *SAMSN1* act as a cytoplasmic adaptor to mediate a signaling pathway
1093	aberrant right subclavian artery (ARSA)	13q31.3(92065636_92299097)x3	233 kb	*ex 2 GCP5*	This gene has been tested for association to diseases (Colitis, Ulcerative; Crohn Disease; Lymphoma). Proteins are expected to have molecular function (heparan sulfate proteoglycan binding) and to localize in various compartments (integral to plasma membrane) extracellular space, anchored to membrane, extracellular region, proteinaceous extracellular matrix)
1165	common arterial trunk (CAT)	9q21.32q21.33(86825588_87161409)x3	335 kb	*SLC28A3*	*SLC28A3* encodes Solute Carrier Family 28 Member 3, which plays a role in multiple cellular processes, including neurotransmission, vascular tone, adenosine concentration in the vicinity of cell surface receptors, and transport and metabolism of nucleoside drugs
1278	abnormal heart rotation	1p36.32(2633351_3161118)x3	522 kb	*ex 1-3 PRDM16*	*PRDM16* acts as a transcription coregulator that controls the development of brown adipocytes in brown adipose tissue. The protein encoded by this gene is a zinc finger transcription factor. *PRDM16* controls the cell fate between muscle and brown fat cells
1280	atrioventricular septal defect (AVSD)	2q14.2(121549137_121659393)x3	110 kb	*GLI2*	Heterozygous mutation in the *GLI2* gene was described in patients with Culler–Jones syndrome
2093	Ebstein Syndrome	10q26.12(122509983_122668106)x3	158 kb	*WDR11*	WDR11 is a member of the WD repeat-containing protein family. Heterozygous mutation in the *WDR11* gene was described in patients with congenital idiopathic hypogonadotropic hypogonadism (IHH).

## Data Availability

Submission of the microarray data to databases may be problematic for ethical reasons, but the data are available upon request to the corresponding author in compliance with EU GDPR.
